# Trait Disinhibition and NoGo Event-Related Potentials in Violent Mentally Disordered Offenders and Healthy Controls

**DOI:** 10.3389/fpsyt.2020.577491

**Published:** 2020-12-11

**Authors:** Carl Delfin, Emily Ruzich, Märta Wallinius, Malin Björnsdotter, Peter Andiné

**Affiliations:** ^1^Centre for Ethics, Law and Mental Health, Department of Psychiatry and Neurochemistry, Institute of Neuroscience and Physiology, Sahlgrenska Academy, University of Gothenburg, Gothenburg, Sweden; ^2^Research Department, Regional Forensic Psychiatric Clinic, Växjö, Sweden; ^3^MedTech West, Sahlgrenska University Hospital, Gothenburg, Sweden; ^4^Lund Clinical Research on Externalizing and Developmental Psychopathology, Child and Adolescent Psychiatry, Department of Clinical Sciences Lund, Lund University, Lund, Sweden; ^5^Affective Psychiatry, Sahlgrenska University Hospital, Gothenburg, Sweden; ^6^Forensic Psychiatric Clinic, Sahlgrenska University Hospital, Gothenburg, Sweden; ^7^Department of Forensic Psychiatry, National Board of Forensic Medicine, Gothenburg, Sweden

**Keywords:** trait disinhibition, event-related potential, ERP, N2, P3, mentally disordered offenders, forensic psychiatry

## Abstract

Trait disinhibition may function as a dispositional liability toward maladaptive behaviors relevant in the treatment of mentally disordered offenders (MDOs). Reduced amplitude and prolonged latency of the NoGo N2 and P3 event-related potentials have emerged as promising candidates for transdiagnostic, biobehavioral markers of trait disinhibition, yet no study has specifically investigated these two components in violent, inpatient MDOs. Here, we examined self-reported trait disinhibition, experimentally assessed response inhibition, and NoGo N2 and P3 amplitude and latency in male, violent MDOs (*N* = 27) and healthy controls (*N* = 20). MDOs had a higher degree of trait disinhibition, reduced NoGo P3 amplitude, and delayed NoGo P3 latency compared to controls. The reduced NoGo P3 amplitude and delayed NoGo P3 latency in MDOs may stem from deficits during monitoring or evaluation of behavior. NoGo P3 latency was associated with increased trait disinhibition in the whole sample, suggesting that trait disinhibition may be associated with reduced neural efficiency during later stages of outcome monitoring or evaluation. Findings for NoGo N2 amplitude and latency were small and non-robust. With several limitations in mind, this is the first study to demonstrate attenuated NoGo P3 amplitude and delayed NoGo P3 latency in violent, inpatient MDOs compared to healthy controls.

## Introduction

Mentally disordered offenders (MDOs) demonstrate a variety of mental disorders, including schizophrenia spectrum disorders, substance abuse, and personality disorders, and pose significant challenges to the criminal justice system, with complex treatment needs and security concerns (1, 2). It is currently believed, however, that MDOs are involved in crime not primarily due to their mental disorders, but due to the same, general risk factors that are present in offenders without mental disorders (3). Indeed, an antisocial personality pattern characterized by poor self-control, restlessness, and impulsivity appears to be the most relevant clinical risk factor for recidivism in MDOs (4), and addressing this pattern should be a primary focus in treatment. Unfortunately, actual evidence specifically addressing treatment of MDOs is lacking, and although guidelines exist (5), there is an urgent need for more research in all areas of intervention (6, 7).

The frequent co-occurrence of disorders on the so-called externalizing spectrum, including ADHD, conduct disorder, antisocial personality disorder, and substance use disorders, is thought to reflect a heritable dispositional liability toward deficient impulse control (8–10). This broad dispositional liability, termed externalizing proneness or *trait disinhibition*, manifests behaviorally as poor self-control, irresponsibility, impatience, recklessness, insistence on immediate gratification, aggression, and engagement in various antisocial activities (11). Since these behavioral manifestations of trait disinhibition are important risk factors for recidivism in MDOs, it may be more fruitful for treatment efforts to target the continuously distributed trait disinhibition construct—representing the shared variance between the aforementioned behaviors—rather than addressing specific externalizing disorders individually (8).

Trait disinhibition may be described as a biobehavioral liability toward maladaptive behaviors that can be assessed using different modalities, including self-report instruments, behavioral task performance, and neurophysiological measurements (12). One such self-report instrument, the Externalizing Spectrum Inventory-Brief Form (ESI-BF), was developed specifically to facilitate efficient assessment of trait disinhibition (13). Importantly, the ESI-BF contains a subscale termed General Disinhibition (henceforth ESI-BF_DIS_) which contains no alcohol or drug-related items and no aggression-related items, thus offering a precise self-report assessment of the trait disinhibition construct.

Behavioral tasks that assess response inhibition, a core executive function necessary to suppress impulsive, routine, and automatic behaviors (14), are often used to probe the trait disinhibition construct. A wealth of research has shown that deficient response inhibition—that is, a reduced ability to inhibit prepotent responses—is robustly associated with externalizing spectrum disorders (15–17). Moreover, the association appears strongly genetically mediated, suggesting that both impaired response inhibition and externalizing disorders stem from the same trait disinhibition construct (10).

In addition to self-report instruments and behavioral tasks, event-related potentials (ERPs), providing a direct measurement of the brain's post-synaptic neural activity, may be used to bridge the gap between brain and behavior. Reduced amplitude of the P3 ERP—a positive voltage deflection peaking around 300 ms after stimulus onset—has emerged as a particularly promising, heritable biobehavioral marker of behaviors characterized by trait disinhibition (18–20). However, although previous research has established P3 amplitude reduction as robustly associated with externalizing disorders and antisocial behavior, studies on MDO samples remain scarce (21).

Going beyond P3 amplitude reduction, research suggests that the amplitude and latency of the P3 ERP may represent distinct endophenotypes. Whereas amplitude is thought to reflect the amount of cognitive resources or “neural power” being used, latency, often defined as the time point where the waveform peaks, may serve as an index of neural processing speed or “neural efficiency” (22, 23). Studies investigating P3 latency are limited, especially in antisocial populations, and results have been mixed. For instance, studies comparing violent and non-violent offenders have found evidence of both delayed P3 latency (24) and no differences in P3 latency (25). Similarly, whereas one study found that reduced P3 amplitude was associated with externalizing in youths, with no effect of P3 latency (26), another study found delayed P3 latencies in young offenders with conduct disorder symptoms compared to healthy controls (27). Delayed P3 latencies have also been associated with higher levels of aggression and hostility (28), and recent meta-analytic research found evidence of prolonged NoGo P3 latencies in ADHD, although findings remain inconclusive, both in offender and in non-offender samples (19, 29).

Apart from the distinction between amplitude and latency, different subcomponents of the P3 ERP are observed depending on the type of behavioral task used. Most ERP research in antisocial populations has focused on the so-called P3a or P3b components. The frontally distributed P3a, or “novelty P3,” is elicited by task-irrelevant infrequent stimuli, whereas the more parietally distributed P3b is associated with the detection of task-relevant infrequent stimuli. In contrast, infrequent stimuli that require response withholding—as in response inhibition tasks—elicits the frontally distributed NoGo P3 component (30). Although still debated, the NoGo P3 is thought to reflect processes related to monitoring and outcome evaluation (31–33). Longitudinal studies in children (34, 35) and young adults (36) have demonstrated that both P3a and P3b amplitude reduction is predictive of a wide range of behaviors characterized by trait disinhibition, including aggression and criminality. These findings are corroborated by meta-analytic studies showing that reduced amplitude and, although less consistently, longer latency of the P3a (21) and P3b (37) is associated with antisocial behavior. However, the bulk of previous research has focused on the P3a and P3b components. While there is emerging evidence suggesting that reduced amplitude of the NoGo P3 component is also associated with externalizing behavior (18, 20, 38–41), so far, no study has been done on an inpatient MDO sample.

Another ERP worth exploring is the N2. The N2 is a negative voltage deflection peaking around 200 ms after stimulus onset. As with the P3, different subcomponents are elicited depending on task and stimuli (42). The NoGo N2 is thought to reflect either the inhibition of a premature response plan, or the detection or resolution of a response conflict between executing and withholding a response (32, 33). Reduced NoGo N2 amplitude has been associated with poor response inhibition (43), alcohol abuse (44), ADHD (45), and impulsive-violent offending (46), rendering it a promising biobehavioral marker of trait disinhibition. Indeed, a recent meta-analysis found that reduced amplitude of the N2 ERP is robustly associated with antisocial behavior (47), although with inconclusive findings regarding psychopathic and subclinical, impulsive samples. A potential explanation, the authors suggest, is that N2 amplitudes may be reduced only at the severe end of the externalizing spectrum (i.e., in violent and antisocial individuals), and thus not necessarily in impulsive, but subclinical, samples, who may be able to recruit compensatory mechanisms. With regards to N2 latency, results have been more inconsistent. For instance, some studies report delayed N2 latencies in individuals with poor response inhibition (43) and alcohol dependence (48), while other studies, although observing reduced amplitudes, saw no concurrent delays in latency in individuals with alcohol abuse (44) and ADHD (45). Thus, while the N2 component may be a relevant biobehavioral marker for trait disinhibition, the inconsistency of previous findings warrants further research.

In sum, trait disinhibition is thought to reflect a dispositional liability toward a broad range of maladaptive behaviors characterized by impulse control problems that are relevant in forensic mental health settings. Reduced amplitude and prolonged latency of the N2 and P3 ERPs have emerged as promising candidates for transdiagnostic, biobehavioral markers of trait disinhibition, yet some important issues remain unresolved. First and foremost, studies in MDO samples remain scarce. Since MDOs are characterized by heterogeneity in both demographics, mental disorders, clinical needs, pharmacological treatment, criminal behaviors, and risk factors for recidivism (49–51), the scarcity of research in MDO samples makes it difficult to evaluate whether the N2 and P3 ERPs are reliable and useful in forensic mental health settings. Furthermore, while P3 amplitude reductions in externalizing and antisocial individuals are well-documented, most P3 research has focused on the P3a or P3b components. As emphasized in recent research (21), the NoGo P3 may be used to further test the “frontal dysfunction hypothesis” of antisocial behavior, which posits the existence of both structural and functional impairments in the prefrontal brain regions of impulsive antisocial individuals (52, 53).

The current study aimed to address these unresolved issues by investigating trait disinhibition using three different modalities, utilizing both between-group comparisons and a whole-sample correlation approach, in a sample of male, violent, inpatient MDOs and healthy controls. Specifically, we used the ESI-BF_DIS_ as a self-report measure of trait disinhibition, and a Go/NoGo task combined with electroencephalography (EEG) to elicit NoGo N2 and P3 ERPs. We hypothesized that MDOs would present with increased levels of self-reported trait disinhibition, reduced response inhibition accuracy, reduced NoGo N2 and P3 amplitudes, as well as prolonged NoGo N2 and P3 latencies, compared to healthy controls. Further, we hypothesized that NoGo N2 and P3 amplitudes would be negatively correlated with self-reported trait disinhibition and positively correlated with response inhibition accuracy, and that NoGo N2 and P3 latencies would be positively correlated with self-reported trait disinhibition and negatively correlated with response inhibition accuracy.

## Methods

All computational materials used in this study are described and publicly available at https://osf.io/yscdh/ (doi: 10.17605/OSF.IO/YSCDH).

### Participants

Inpatient MDOs were recruited following completion of a parallel, ongoing study at a maximum security forensic psychiatric hospital in Sweden. All MDOs, following completion of the parallel study, were asked to participate in the current study if they were male and had, at any point, been sentenced for a violent crime, regardless of their psychiatric diagnosis. Exclusion criteria were a history of brain damage with lasting effects and if the treating psychiatrist deemed participation unsuitable due to current psychiatric status or safety concerns. A total of 29 MDOs agreed to participate. One MDO was removed from data analysis due to having less than four correct NoGo trials left after EEG preprocessing, and one MDO was excluded due to missing all self-report data. One MDO lost approximately one third of the EEG data due to technical issues during recording, although remaining data was retained for analysis. Thus, data from a total of 27 MDOs were available for analysis in the current study. All MDOs participated while on their usual medication (Table 1) and treatment plan, and had been sentenced to forensic psychiatric care with a so-called “special court supervision,” a form of supervision used in cases where there is a substantial risk of relapse into serious criminality due to severe mental disorder (54). All MDO participants received a voucher (~$10) for use either in the hospital's kiosk or at a local mall as reimbursement directly after completed participation.

**Table 1:** Overview of pharmacological treatment in the MDO group (*N* = 27).

**Pharmacological category**	***N* (%)**
ADHD substances	3 (11%)
Anticholinergics	14 (52%)
Antidepressants	7 (26%)
Antiepileptics	5 (19%)
Antipsychotics	20 (74%)
Benzodiazepine sedatives/hypnotics	5 (19%)
Non-benzodiazepine sedatives/hypnotics	9 (33%)
Somatic substances	14 (52%)
SUD substances	1 (4%)

The control group consisted of healthy male volunteers recruited, using posters, e-mail, and verbal information, from staff at two hospitals as well as from students at a university. Exclusion criteria were having completed higher education (i.e., having received a degree after 3 years of higher education or more), having a history of brain damage with lasting effects, having a current major mental disorder, and drug use within the last 6 months. A total of 25 control group participants were recruited. Five control group participants had to be excluded after participation but prior to data analysis due to reporting drug use within the last 6 months prior to participation and/or having a history of brain damage with lasting effects in the self-report questionnaires. As such, data from 20 healthy controls was available for analysis. Control group participants received either a voucher (~$10) for use at a local mall or a movie ticket as compensation for the time spent in the study.

All participation was voluntary and based on informed, written consent. Data collection took place between May, 2017 and March, 2019.

### Measures

The MDO group completed a large battery of questionnaires during participation in the parallel study, and completed one additional questionnaire prior to EEG acquisition. The control group answered a battery of questionnaires consisting of demographic questions (e.g., age, educational level), questions related to exclusion criteria, and several self-report instruments. The current study analyses data from the trait disinhibition questionnaire only.

#### Trait Disinhibition

Trait disinhibition was quantified using the ESI-BF_DIS_. The ESI-BF (13) is a shorter version of the Externalizing Spectrum Inventory (ESI) (9), developed to provide a self-report measure of a wide range of disinhibitory behaviors. The ESI-BF was co-translated into Swedish by two of the authors of the current study (CD and MW) and consists of 160 items rated from 0 (Not true at all) to 3 (Completely true). Scores on the items may be summed to three subscales: General Disinhibition, Callous-Aggression, and Substance Abuse. In the current study, only the 20 item ESI-BF_DIS_ subscale was used, with possible scores ranging from 0 to 60. The ESI-BF_DIS_ subscale includes items related to irresponsibility, impulsiveness, recklessness, as well as robbery, theft, and exploiting others for their own gain. Following recent recommendations (55), internal reliability was assessed using both Cronbach's alpha and McDonald's Omega. The ESI-BF_DIS_ showed high internal reliability in the current study, with Cronbach's alpha = 0.91 and McDonald's Omega total = 0.93, similar to or above what has been reported in previous community (56–58), prison (58), and MDO samples (57).

#### Response Inhibition Task

**Figure 1:**
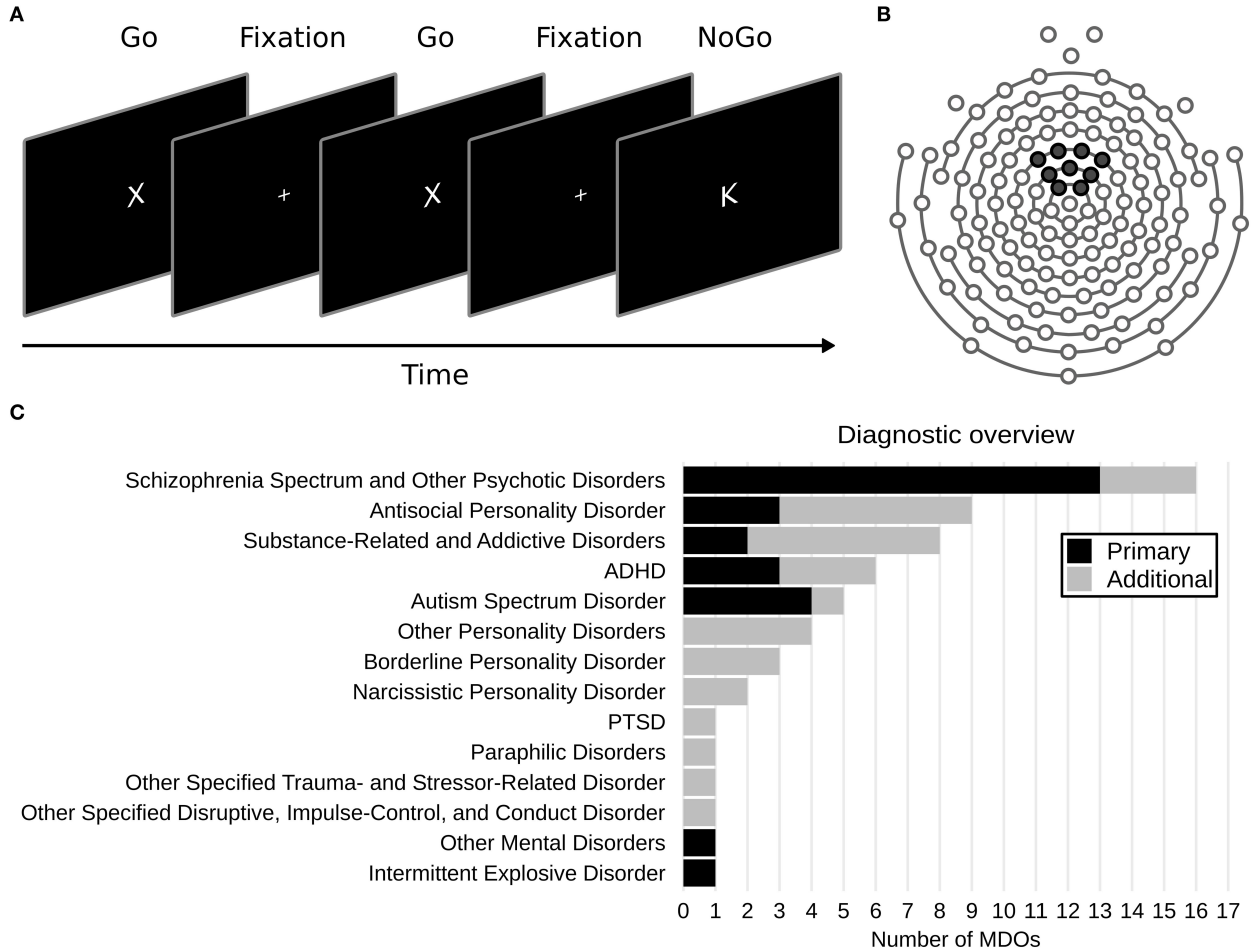
**(A)** Overview of the Go/NoGo task. **(B)** Layout of the 128 channel sensor net used. Black markers show the location of the nine electrodes used to create a frontocentral region of interest. **(C)** Overview of DSM-5 diagnoses in the mentally disordered offenders (MDOs) group.

A Go/NoGo task based on previous work (59, 60) and implemented using Presentation (Neurobehavioral Systems Inc.) was used to assess response inhibition and elicit ERPs. Participants were seated comfortably ~60 cm from the monitor (a 22 inch widescreen LCD TFT monitor, with a 1680 × 1050 pixel resolution running at 60 Hz) used to display task stimuli. They were instructed to respond as quickly and accurately as possible by pressing the left mouse button every time a Go stimulus (a white “X”) appeared, and to withhold a response whenever a NoGo stimulus (a white “K”) appeared. The Go and NoGo stimuli (“X” and “K”) were presented for 250 ms in white text against a black background, with a fixation point (+) shown between trials, located at the center of the screen (Figure 1A). The Helvetica font (size 14) was used for all stimuli. The inter-stimulus interval was pseudorandomly distributed between 1,000 and 3,000 ms at 500 ms increments. Prior to beginning the task, participants were given oral and written instructions, and then completed 10 practice trials requiring at least 50% correct responses. If <50% were correct, 10 new trials began, and so on, until the threshold for correct response was met. The task consisted of 326 trials, of which 274 (84%) were Go trials and 52 (16%) were NoGo trials, divided into two blocks of 162 trials each, with rest in between. Participants could rest however long they wanted. Go trials appeared with higher frequency than NoGo trials to establish a prepotency to respond, thus making it more difficult to inhibit responses. Two participants in the MDO group completed a longer version of the Go/NoGo task, which was since revised due to time constraints. Participants also completed a resting-state task following the Go/NoGo task, the results of which are not reported in the current study.

#### Clinical Characteristics

MDOs' psychiatric diagnoses, criminal history, and pharmacological treatment at the time of the EEG recording were retrieved from medical records at the treatment facility. Diagnoses are presented according to the Diagnostic and Statistical Manual of Mental Disorders, Fifth Edition (DSM-5) (61). Pharmacological treatment was defined as all medication received in the 24 h preceding EEG recording, as well as any acting depot injections, and was coded as either “yes” or “no” for nine major pharmacological categories: antipsychotics, antidepressants, benzodiazepine sedatives/hypnotics, non-benzodiazepine sedatives/hypnotics, anticholinergics, antiepileptics, ADHD substances, SUD substances, and somatic substances.

### Procedure

#### Ethics

This study was approved by the regional ethics review board in Linköping, Sweden (2017/56-31, 2018/7-32, 2018/321-32), and was conducted in accordance with the Declaration of Helsinki, including all participants providing voluntary, informed consent prior to participation.

#### EEG Data Acquisition

EEG was recorded using two computers and a high-impedance NetStation NA400 amplifier (Electrical Geodesics Inc.). One computer was used to deliver stimuli, record responses, and send digital triggers to the acquisition computer. The acquisition computer was connected to the amplifier and received digital triggers and EEG signals. EEG signals were recorded using 128 Ag/AgCl electrodes positioned in a Hydrocel Geodesic sensor net, using Net Station software (Electrical Geodesics Inc.), sampled at 1,000 Hz and referenced to the midline vertex electrode (Cz) during recording. Impedances for each electrode were kept below 100 KΩ when possible, although this was not achieved for all participants due to, e.g., time constraints. Since all recordings took place in cool and dry recording environments along with state-of-the-art filtering, artifact detection, and artifact rejection methods, high impedances should not have had a considerable impact on our results (62). The EEG acquisition procedure, including preparation, the Go/NoGo task, and an additional resting-state task, took ~45 min for each participant.

#### EEG Data Preprocessing

The EEG preprocessing pipeline was carried out using version 0.20.4 of the MNE-Python module (63, 64) running on Python 3.8.2, and largely adhered to current recommended procedures to ensure quality and reproducibility (65, 66). Apart from the removal and interpolation of bad channels, independent component analysis (ICA) components, and artifacts following visual inspection, the entire EEG data preprocessing pipeline was automated in order to maintain consistency and remove bias (all code is available online at the Open Science Framework; https://osf.io/yscdh/). Briefly, the raw EEG data was first visually inspected and bad channels marked. Data were then bandpass filtered from 0.1 to 30 Hz using current MNE recommendations (finite impulse response filter with zero phase and a Hamming windowed design) and bad channels were interpolated. Following interpolation, ICA was used to identify and remove artifacts. Since ICA can be sensitive to low frequency drifts, a copy of the raw data was bandpass filtered at 1 to 30 Hz and used to find ICA components. All ICA components were visually assessed and components representing eye blinks, saccades, heartbeats, and other non-brain related signals were marked and zeroed out from the raw data that was filtered using a 0.1 to 30 Hz bandpass. The data was then epoched around a stimulus-locked window of 1,000 ms (−200 to 800 ms after stimulus presentation) and baseline corrected (-200 to 0 ms). Finally, automated artifact rejection using Autoreject version 0.2.1 (67), with default values for peak-to-peak thresholding, was used to interpolate artifactual channels and remove contaminated epochs. There were no robust differences between MDOs and controls in the number of bad channels removed and interpolated, number of ICAs zeroed out, and number of correct NoGo trials left after artifact rejection (Supplementary Table 1). Preprocessed data was saved as comma separated values and imported into R for statistical analysis.

#### Statistical Analysis

Only non-subtracted, correct NoGo trials were analyzed in the current study (32). Since ERP measurements are more reliable when data is averaged across multiple electrodes (20, 68), all ERPs were based on a frontocentral region of interest (ROI) created by averaging data from nine electrodes (E20, E12, E5, E118, E13, E6, E112, E7, E106; Figure 1B). NoGo N2 amplitude was defined as the mean amplitude in the 225 to 325 ms window post-stimuli (NoGo N2_WIN_) and NoGo P3 amplitude was defined as the mean amplitude in the 325 to 625 ms window post-stimuli (NoGo P3_WIN_). The windows were chosen to avoid overlap between components, and are comparable to windows used in previous studies on forensic and antisocial participants (18, 38, 40, 69). Latency was defined as the 50% fractional area latency for each ERP, which is the time point before which 50% of the negative (for N2) or positive (for P3) area of the waveform is observed (70). Furthermore, in order to account for potential latency effects, we also calculated moving window amplitudes, defined as the mean amplitude 50 ms before and 50 ms after the 50% positive fractional area latency was observed (NoGo N2_MOV_ and NoGo P3_MOV_).

All statistical analysis was carried out using the R statistical language, version 4.0.0 (71). Several packages from the Tidyverse (72) were used for intermediate data processing and plotting. We employed a fully Bayesian approach, and all statistical models were specified using the R package brms (73), which provides an interface to the Stan probabilistic programming language (74). All Bayesian priors were chosen to be robust and weakly informative, thus having negligible impact on obtained estimates while providing moderate regularization of potential outliers (75). Group differences were modeled using a robust linear regression approach using a Student's T distribution (76) while allowing unequal variances between groups, and correlations were modeled using a multivariate Student's T distribution with the correlation matrix drawn from an LKJ (2) prior (77). Model sampling was carried out using 8 chains with 4,000 iterations each, after 1,000 warmup iterations were discarded. All models were diagnosed using the R package ShinyStan by screening traceplots for convergence, ensuring that Gelman-Rubin diagnostics (R-hat) were <1.1, that the number of effective samples were > 10% of the total sample size, and that there were no issues with autocorrelation.

From group comparisons we report the estimated posterior medians of group differences and bias-corrected standardized group differences (MDOs relative to controls). Note that there are several standardized difference statistics (i.e., effect size statistics), and unfortunately, different authors use different notations and symbols. In light of this discrepancy, we follow recommendations (78) and use $\hat{\delta }$ to denote what is often referred to as Cohen's *d* with Hedges's *g* correction (see Supplementary Materials for the exact equations used). Median values are presented along with 90% highest density intervals (HDIs; presented within square brackets). The 90% HDI may be interpreted such that there is a 90% probability—or, in non-mathematical terms, very likely (79)—that the estimate falls within its range. From correlation analyses, we report the median estimated correlation coefficient (ρ) along with 90% HDIs. For figures, we also plot the 66% HDI, which may be interpreted as a “likely region” (79). Finally, for readers unfamiliar with the Bayesian framework, we also report the probability of direction (PD), which is defined as the probability that an effect is of the same sign as the median's (80, 81). The PD ranges from 50 to 100% and may be interpreted as the probability that an effect exists (i.e., that the estimate is different from zero). It has a 1:1 correspondence to the frequentist *p*-value, such that $p_{two-tailed}=2\left(1-\;\frac{PD}{100}\right)$.

#### Reliability Analysis

To ensure that Go/NoGo task performance and ERP measurements were reliable, we calculated the split-half reliability by estimating correlations between the averages of odd and even trials (12, 20), corrected using the Spearman-Brown formula (82) and denoted ρ_SB_. The reliability of the Go/NoGo paradigm was high, with ρ_SB_ = 0.79 [0.65, 0.89] for median NoGo response time and ρ_SB_ = 0.89 [0.84, 0.94] for NoGo accuracy. With the exception of NoGo N2 latency, which showed only moderate reliability (ρ_SB_ = 0.62 [0.39, 0.78]), the reliability of all ERP measurements was high, with ρ_SB_ = 0.78 [0.62, 0.89] for NoGo N2_WIN_, ρ_SB_ = 0.77 [0.60, 0.88] for NoGo N2_MOV_, ρ_SB_ = 0.82 [0.71, 0.91] for NoGo P3_WIN_, ρ_SB_ = 0.91 [0.85, 0.95] for NoGo P3_MOV_, and ρ_SB_ = 0.81 [0.68, 0.90] for NoGo P3 latency.

## Results

### Demographics and Clinical Characteristics

Participants (*N* = 47) were on average 35.13 years old (*SD* = 10.76, range = 20–58). MDOs (*N* = 27) were ~ 4 years older than controls (*N* = 20), with an average of 36.63 years (*SD* = 9.85, range = 20–57) for MDOs and 33.10 years (*SD* = 11.82, range = 20–58) for controls. The estimated difference was 3.96 [−1.64, 9.6], with a small to moderate effect size ($\hat{\delta }$ = 0.37 [−0.14, 0.90]) and PD = 88%. The most frequent level of education in both groups was having finished high school (60% in the control group and 44% in the MDO group), although four participants in the MDO group (15%) had not finished primary school.

Overall, MDOs presented with a wide range of mental disorders. The most common primary diagnosis was schizophrenia spectrum and other psychotic disorders (*N* = 13, 48%), while the most common additional diagnoses were antisocial personality disorder (*N* = 6, 22%) and substance-related and addictive disorders (*N* = 6, 22%). Most MDOs had one additional diagnosis (i.e., median *N* = 1), ranging from 0 to 4 (see Figure 1C for an overview), and most MDOs also received three different pharmacological substances (range = 0–6), although four MDOs did not receive any pharmacological treatment at all (see Table 1 for an overview).

The criminal history of the MDOs was heterogeneous, with a mean total number of 7.30 sentences (*SD* = 7.10), ranging from 1 to 31. The mean age at first sentencing was 21.48 years (*SD* = 7.30, range = 15–39; note that 15 is the minimum age for criminal sentencing in Sweden), while the mean age at first reported crime was 13.33 years (*SD* = 4.40, range = 6–26). As per inclusion criteria, all MDOs had at some point been sentenced for a violent crime. Eight MDOs (30%) had committed acts of deadly violence, of which two (7%) repeatedly (i.e., on two or more occasions). A total of 23 MDOs (85%) had committed assault, including aggravated, of which 18 (67%) had done so repeatedly. Finally, six MDOs (22%) had committed sexual crimes, of which five (19%) repeatedly.

### Self-Reported Trait Disinhibition and Go/NoGo Task Performance

**Figure 2:**
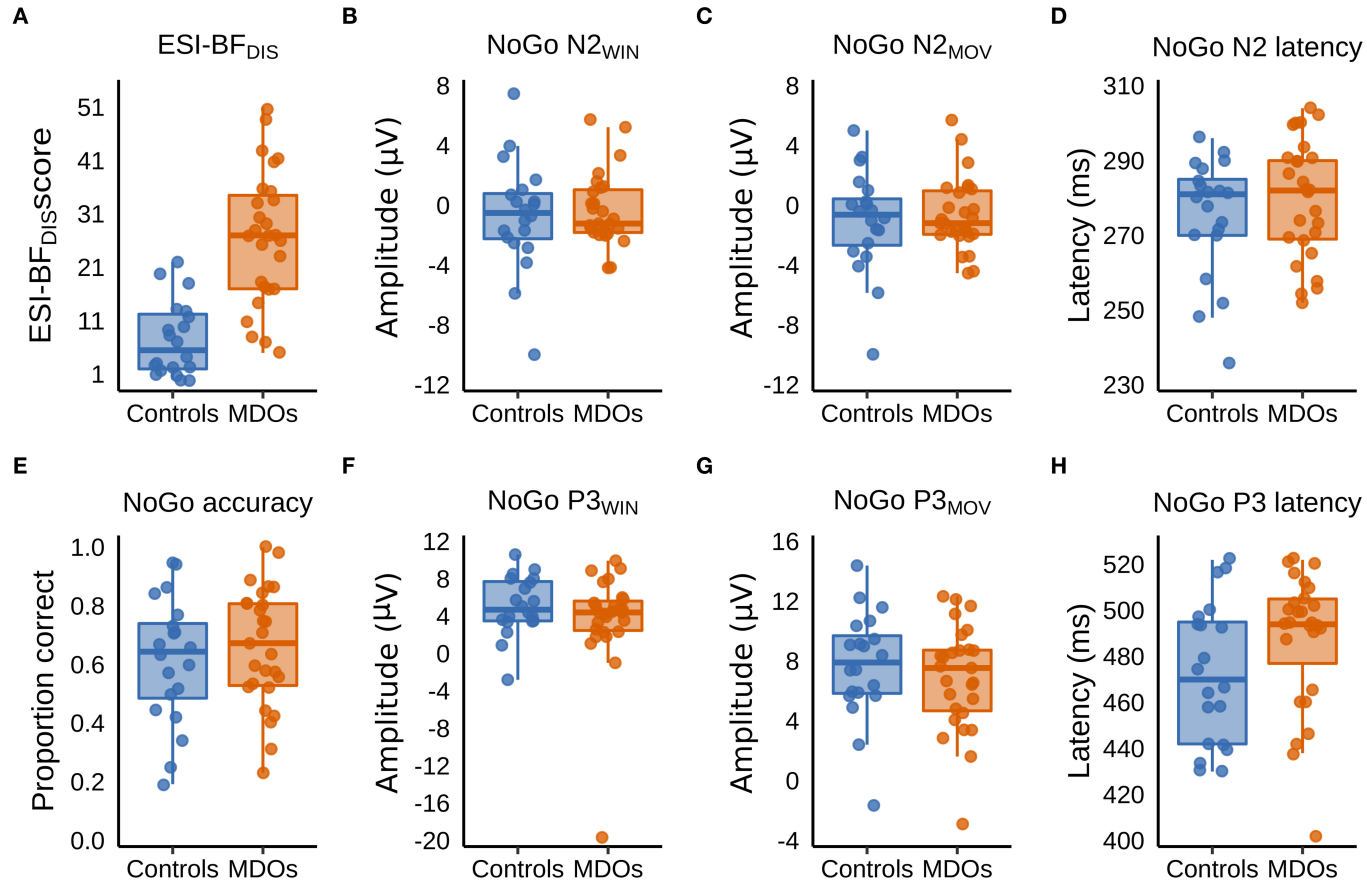
**(A–H)** Boxplots showing group differences in trait disinhibition, experimentally assessed response inhibition, and event-related potentials. ESI-BF_DIS_, Externalizing Spectrum Inventory-Brief Form, General Disinhibition subfactor; MDO, mentally disordered offender.

MDOs presented with a higher degree of self-reported trait disinhibition than controls. The estimated median difference was over 19 points, with posterior estimates showing that, with 90% probability, MDOs scored between 14 and 24 points higher on the ESI-BF_DIS_ than controls, indicating a large and robust difference in self-reported trait disinhibition between the two groups. Contrary to our hypotheses, MDOs were also able to inhibit responses during NoGo trials at a slightly higher rate than controls. With 90% probability, MDOs' NoGo accuracy rate was between 7% lower and 15% higher than controls, indicating a small and non-robust group difference. For details, see Table 2 and Figure 4A. Boxplots are presented in Figure 2.

**Table 2:** Descriptive overview and posterior estimates of group differences.

	**Whole sample (*****N*** **=** **47)**		**Controls (*****N*** **=** **20)**		**MDOs (*****N*** **=** **27)**		**Posterior estimates**		
**Measure**	**Mean ± SD**	**Range**	**Mean ± SD**	**Range**	**Mean ± SD**	**Range**	**Diff. [90% HDI]**	**δ[90% HDI]**	**P_**D**_**
ESI-BF_DIS_	18.49 ± 14.09	0–51	7.5 ± 6.91	0–22	26.63 ± 12.43	5–51	19.37 [14.41, 24.35]	1.83 [1.25, 2.46]	100%
NoGo accuracy	0.64 ± 0.21	0.19–1	0.62 ± 0.21	0.19–0.94	0.66 ± 0.2	0.23–1	0.05 [−0.07, 0.15]	0.22 [−0.31, 0.71]	76%
NoGo N2_WIN_	−0.47 ± 2.97	−9.98–7.47	−0.69 ± 3.64	−9.98–7.47	−0.32 ± 2.42	−4.17–5.75	0.12 [−1.44, 1.61]	0.05 [−0.53, 0.58]	55%
NoGo N2_MOV_	−0.79 ± 2.82	−9.95–5.71	−1.02 ± 3.34	−9.95–5.01	−0.62 ± 2.41	−4.53 – 5.71	0.14 [−1.32, 1.56]	0.05 [−0.51, 0.57]	57%
NoGo N2 latency	277.96 ± 15.77	236–304	275.4 ± 15.92	236–296	279.85 ± 15.68	252–304	3.75 [−4.05, 11.86]	0.24 [−0.27, 0.74]	79%
NoGo P3_WIN_	4.3 ± 4.53	−19.66–10.6	5.09 ± 3.13	−2.79–10.6	3.71 ± 5.32	−19.66–9.94	−0.73 [−2.26, 0.78]	−0.28 [−0.83, 0.31]	79%
NoGo P3_MOV_	7.25 ± 3.53	−2.89–14.39	7.72 ± 3.59	−1.65–14.39	6.90 ± 3.51	−2.89–12.33	−0.82 [−2.62, 0.91]	−0.24 [−0.74, 0.29]	78%
NoGo P3 latency	481.28 ± 30.49	402–522	472.6 ± 30.55	430–522	487.7 ± 29.36	402–522	17.41 [2.23, 32.53]	0.59 [0.06, 1.14]	97%

*ESI-BF_DIS_, Externalizing Spectrum Inventory-Brief Form, General Disinhibition subfactor; MDO, mentally disordered offender; HDI, Highest Density Interval; P_D_, Probability of Direction*.

### NoGo N2

**Figure 3:**
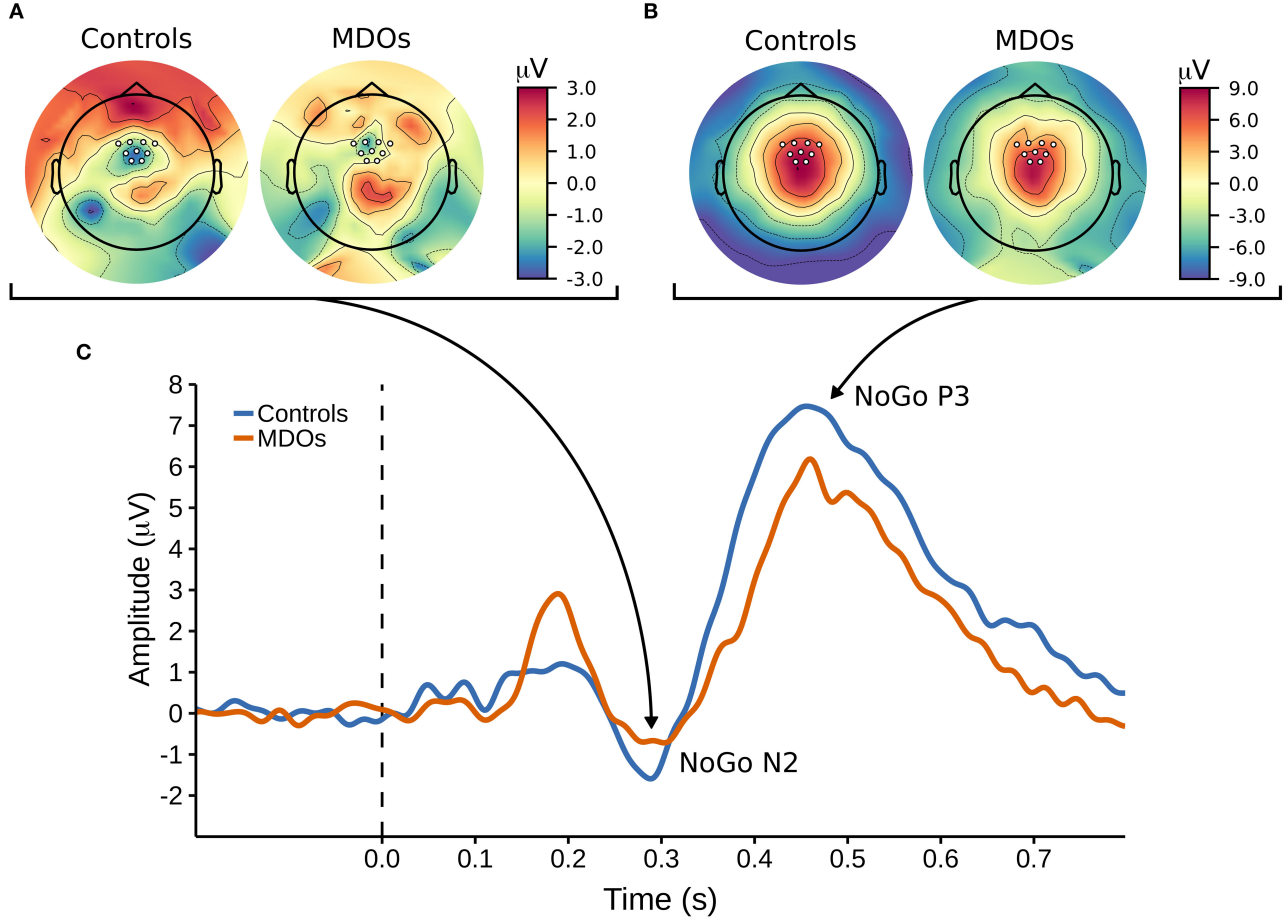
**(A,B)** Topographic plots showing the cortical distribution of amplitude at **(A)** 290 ms post-stimuli and **(B)** 450 ms post-stimulim, with white markers indicating the locations of the nine electrodes used to create a frontocentral region of interest. **(C)** The NoGo ERP waveform, with arrows pointing at corresponding topographic timepoints for the NoGo N2 and NoGo P3 components. MDOs, mentally disordered offenders.

**Figure 4:**
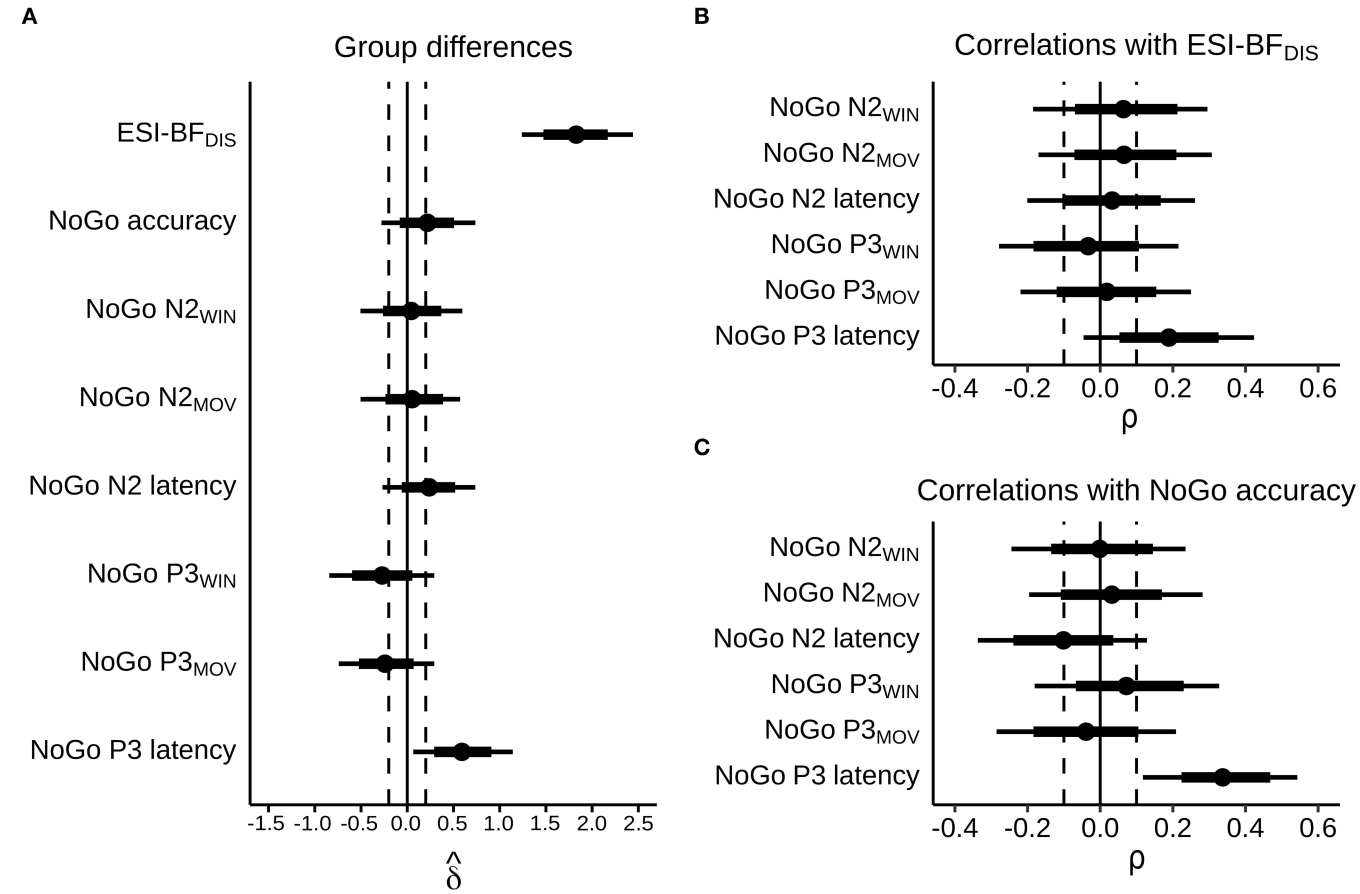
Posterior estimates, with dots representing posterior medians, thick bars representing 66% highest density intervals, and narrow bars representing 90% highest density intervals. Dashed lines indicate cutoffs for “small” effect sizes, corresponding to absolute values of $\hat{\delta }$ = 0.2 and ρ = 0.1. **(A)** Estimated bias-corrected standardized group differences, MDOs relative to controls. **(B)** Estimated correlations with self-reported trait disinhibition in the whole sample. **(C)** Estimated correlations with NoGo accuracy in the whole sample.

Controls had larger (i.e., more negative) NoGo N2 amplitudes than MDOs, although the differences were both small and non-robust, as indicated by negligible effect sizes for both NoGo N2_WIN_ and NoGo N2_MOV_ (both median $\hat{\delta }$ = 0.05) along with wide HDIs. Likewise, NoGo N2 latency was delayed in MDOs, with a median group difference of 3.75 ms, corresponding to a small effect size (median $\hat{\delta }$ = 0.24), but the HDI remained relatively wide. The grand average waveform and associated topographic plots is shown in Figure 3, with full details presented in Table 2 and Figure 4A. Boxplots are presented in Figure 2.

### NoGo P3

MDOs showed both reduced NoGo P3 amplitude and delayed latency compared to controls. MDOs estimated median NoGo P3_WIN_ amplitude was 0.73 μV lower than controls, whereas MDOs estimated median NoGo P3_MOV_ amplitude was 0.82 μV lower than controls, corresponding to small to moderate effect sizes (median $\hat{\delta }$ = −0.28 and −0.24, respectively). MDOs estimated median NoGo P3 latency was 17.41 ms longer than controls, corresponding to a moderate effect size (median $\hat{\delta }$ = 0.59). The grand average waveform and associated topographic plots is shown in Figure 3. Full details are presented in Table 2 and Figure 4A. Boxplots are presented in Figure 2.

### Correlations

We observed a positive, small to moderate, robust correlation between NoGo P3 latency and NoGo accuracy, with ρ = 0.34 [0.12, 0.54], as well as a positive, small albeit relatively less robust correlation between NoGo P3 latency and ESI-BF_DIS_ score, with ρ = 0.19 [−0.05, 0.42]. Thus, longer NoGo P3 latency was associated with better inhibitory control and, although less robustly, with a higher degree of trait disinhibition. Remaining correlations were either negligible (absolute ρ <0.1) or with wide HDIs, indicating high degrees of uncertainty. Details are presented in Table 3 and Figures 4B,C. Scatterplots are presented in Supplementary Figure 1.

**Table 3:** Posterior estimates of correlations.

	**ESI-BF**_*******DIS*******_		**NoGo accuracy**	
**Measure**	**ρ [90% HDI]**	**P_***D***_**	**ρ [90% HDI]**	**P_***D***_**
NoGo N2_WIN_	0.06 [−0.19, 0.29]	67%	0 [−0.25, 0.23]	50%
NoGo N2_MOV_	0.07 [−0.17, 0.31]	67%	0.03 [−0.20, 0.28]	59%
NoGo N2 latency	0.03 [−0.20, 0.26]	59%	−0.10 [−0.34, 0.13]	76%
NoGo P3_WIN_	−0.04 [−0.28, 0.22]	59%	0.07 [−0.18, 0.33]	68%
NoGo P3_MOV_	−0.02 [−0.22, 0.25]	55%	−0.04 [−0.29, 0.21]	60%
NoGo P3 latency	0.19 [−0.05, 0.42]	90%	0.34 [0.12, 0.54]	99%

*ESI-BF_DIS_, Externalizing Spectrum Inventory-Brief Form, General Disinhibition subfactor;HDI, Highest Density Interval; P_D_, Probability of Direction*.

### Exploratory Analyses

Since MDOs had delayed NoGo P3 latencies and, unexpectedly, also responded slightly more accurately on NoGo trials than controls, we explored whether MDOs also had longer median NoGo response times (RTs) than controls, and whether these measures were correlated with median NoGo RT. Note that these analyses were based on data from 20 controls and 26 MDOs, since one participant in the MDO group did not have any failed NoGo trials and thus no data on NoGo RT. MDOs had longer median NoGo RTs than controls, with a median group difference of 9.15 ms [−11.04, 28.53], corresponding to a small to moderate effect size ($\hat{\delta }$ = 0.29 [−0.33, 0.96]). Furthermore, median NoGo RT showed positive, moderate, and robust correlations with both NoGo accuracy (ρ = 0.32 [0.10, 0.55], P_D_ = 98%) and NoGo P3 latency (ρ = 0.38 [0.15, 0.59], P_D_ = 99%).

## Discussion

The current study examined differences in self-reported trait disinhibition, response inhibition, and NoGo N2 and P3 ERPs in male, violent MDOs and healthy controls. In addition, we examined how these different measures were correlated with each other in the whole sample. In line with our hypotheses, we found evidence of higher levels of self-reported trait disinhibition, delayed NoGo P3 latencies, and reduced NoGo P3 amplitudes in MDOs compared to healthy controls. Furthermore, a positive correlation between NoGo P3 latency and self-reported trait disinhibition was observed. These findings suggest that the NoGo P3 ERP—especially NoGo P3 latency—may be feasible as a transdiagnostic, biobehavioral marker of trait disinhibition in MDOs, despite the considerable heterogeneity that characterizes this group, although several caveats are discussed below. In particular, the lack of a non-violent, psychiatric-only comparison group limits the interpretation of our findings. Only small and non-robust differences in NoGo N2 measures were observed, however, and all correlations with NoGo N2 measures were, again, small and non-robust.

As expected, and in line with previous research, MDOs were characterized by trait disinhibition both in a diagnostic sense, with a high prevalence of personality disorders, substance-related and addictive disorders, and ADHD, and in a transdiagnostic, dimensional sense, with substantially higher ESI-BF_DIS_ scores than the control group. A majority of male MDOs in Sweden are diagnosed within the schizophrenia spectrum (54), and this was the case also in the current study. However, the total number (i.e., including additional diagnoses; Figure 1C) of personality disorders, substance-related and addictive disorders, and ADHD diagnoses was twice as high as the total number of schizophrenia spectrum or other psychotic disorders (32 vs. 16 diagnoses in total, respectively). The high prevalence of personality disorders, substance-related and addictive disorders, and ADHD aligns well with the substantially higher scores on the ESI-BF_DIS_ in MDOs compared to controls, offering preliminary support for the use of the ESI-BF_DIS_ as a reliable measure of trait disinhibition in Swedish forensic mental health settings, although proper validation studies are still required. Notably, MDOs scored ~19 points higher than controls on the ESI-BF_DIS_ in the current study, which is higher than the 15 point difference between Dutch MDOs and community volunteers recently reported previously (57). Our findings in conjunction with prior research further highlight the importance of addressing behaviors characterized by trait disinhibition in MDOs, both in research and in treatment, and perhaps especially in recidivism prevention (3, 4).

Surprisingly, MDOs had an estimated accuracy rate of ~5% higher than controls. While this finding must be cautiously interpreted due to the relatively wide HDI and the small effect size, some discussion is warranted, since the effect was opposite to what was expected. The finding that MDOs' had a slightly better accuracy rate than controls, along with the finding of a robust, positive correlation between NoGo P3 latency and NoGo accuracy, prompted us to perform additional exploratory analyses. These analyses showed that MDOs had longer median NoGo RTs (i.e., median response time on *failed* NoGo trials) than controls, and revealed positive correlations between median NoGo RT and both NoGo P3 latency and NoGo accuracy (which are measured on *successful* NoGo trials). Since research suggests that the amplitude and latency of the P3 ERP may represent distinct endophenotypes (22), the positive correlation between NoGo P3 latency and median NoGo RT implies that less efficient neural information processing may attenuate the prepotency to quickly respond on NoGo trials. This attenuated prepotency to quickly respond could, in theory, result in higher NoGo accuracy, which was confirmed by the positive correlation between NoGo accuracy and median NoGo RT found in our exploratory analyses. Taken together, these findings indicate that the slightly higher accuracy rate and longer median NoGo RTs observed in the MDO group may in fact be related to less efficient neural information processing. That is, slower neural information processing—as evidenced by MDOs' longer NoGo P3 latency—may attenuate the prepotency to quickly respond on NoGo trials, which inadvertently leads to better NoGo accuracy. While interesting, this theory remains speculative and based on exploratory analyses, and should be further investigated in larger samples, preferably also comparing MDOs with a non-violent, psychiatric-only sample. For future research, it would be interesting to examine the effects of shorter inter-stimulus intervals. It is also possible that the observed difference was due to chance alone, and that we did not observe more robust group differences in NoGo accuracy because the Go/NoGo paradigm was too easy. A more demanding task, or a task with non-neutral stimuli, might have rendered different results, and should be further explored in future studies. Another critique against the Go/NoGo task is that performance on the task is substantially different from performance in real-world situations where response inhibition is required (14). Still, the use of a more complex task introduces additional confounding variables, takes more time to prepare, and is more difficult to perform, and thus might not be feasible in an MDO population.

Both the observed group differences in NoGo N2_WIN_ and NoGo N2_MOV_, as well as the correlations between these measures and ESI-BF_DIS_ scores and NoGo accuracy, were non-robust with negligible to small effect sizes. A more robust group difference in NoGo N2 latency—but with practically non-existent correlations—was observed, with MDOs demonstrating delayed NoGo N2 latency compared to controls. While this finding is in agreement with previous studies suggesting that delayed NoGo N2 latency may serve as a marker of impulsivity (83) and self-reported psychopathic traits (41), we did not observe any robust correlations between NoGo N2 latency and self-reported trait disinhibition, nor between NoGo N2 latency and response inhibition accuracy. Combined with the relatively low reliability of this ERP component, our findings remain difficult to evaluate. The use of a Go/NoGo task may offer some clues, however. Recent meta-analyses suggest that N2 amplitude reductions are systematically observed primarily in studies using the Stop-Signal Task (47, 84). The Stop-Signal Task is a measure of response cancellation, whereas the Go/NoGo is a measure of response inhibition, and while the two measures are similar they are not identical (85). Future studies should bear this in mind, and preferably compare results from both tasks. Another possibility is that if the NoGo N2 reflects conflict monitoring, these processes may remain intact in MDOs, while later, evaluative processes reflected in the NoGo P3 are diminished.

To the best of our knowledge, this is the first study to demonstrate reduced NoGo P3 amplitudes and delayed NoGo P3 latencies in violent, inpatient MDOs compared to healthy controls. Although our findings do exhibit uncertainty in the form of relatively wide HDIs—likely due to the small sample size—the Bayesian analyses demonstrated with relatively high probability (79% and 78%) that MDOs' NoGo P3 amplitude was lower than controls, and with high probability (97%) that MDOs' NoGo P3 latency was longer than controls. As a safeguard against potential latency differences between the two groups, we quantified amplitude using both a standard fixed window approach as well as a moving window approach. Since the group difference in NoGo P3 amplitude was similar for both NoGo P3_MOV_ and NoGo P3_WIN_, the later peaking of the NoGo P3 latency in MDOs should not have artifactually driven the lower amplitudes among MDOs. Taken together, these findings align with previous research showing reduced NoGo P3 amplitude in antisocial samples (38–41) and, to some extent, individuals with schizophrenia (86). In addition, the estimated median effect sizes in the current study were larger than previous meta-analytic research showing reduced P3 amplitudes (*d* = 0.25) and delayed P3 latencies (*d* = 0.13) in antisocial samples (37) (note that the authors use positive effect sizes to indicate both reduced amplitude and delayed latencies in antisocial individuals compared to controls), as well as comparable to more recent work showing reduced NoGo P3 amplitudes (*d* = −0.57) and delayed NoGo P3 latencies (*d* = 0.35) in individuals with ADHD (87).

Since the NoGo P3 component is thought to reflect outcome monitoring or outcome evaluation after successful inhibitions (31–33), with amplitude reflecting the amount of cognitive resources used and latency serving as an index of neural efficiency (22, 23), it is possible that MDOs have deficits both in the allocation of cognitive resources during monitoring or evaluation of their behavior, as well as in the efficiency of such allocation (88). If monitoring and evaluation capabilities are reduced, it may be difficult to correctly interpret the environment and evaluate possible consequences of one's behavior. Supporting this view, previous research has demonstrated that increased NoGo P3 amplitude predicts more self-control in a social decision-making task, suggesting that deficits in the neural correlates of outcome monitoring/evaluation may lead to adverse outcomes in social contexts (89).

Substantial efforts have been made to pinpoint the neural generators of the NoGo N2 and NoGo P3 ERPs, although findings remain inconclusive. Since successful inhibition during the Go/NoGo task results in increased hemodynamic blood flow across several regions, including the superior, middle, and inferior frontal gyri, the cingulate and insular cortices, as well as temporal, parietal, and occipital areas (90), it seems unlikely that NoGo ERPs are generated by a single source. Indeed, converging evidence suggests that the NoGo N2 and P3 components reflect the sum of several, concurrently activated neural networks (91). Interestingly, however, both components can be traced to the midcingulate cortex (MCC), with contributions from the precentral, middle frontal, inferior frontal, and insular cortices (91–95). It was recently suggested that these contributing regions reflect top-down selective attention rather than response inhibition, positioning the MCC as the primary source of inhibitory processing (96). Corroborating these findings are studies placing the neural generator of the NoGo P3 in the dorsal anterior cingulate cortex [dACC; (97–99)], a region which largely corresponds to the MCC [and, to add further confusion, which is sometimes also referred to as the caudal ACC; for thorough reviews, see (100, 101)]. The exact functions of the MCC/dACC region are not known, although it contributes to both cognitive control and decision making (102), including conflict monitoring and outcome evaluation (103, 104). Interestingly, the gyral regions of the MCC are thought to play an important role in social cognition and social behavior, especially in predicting and monitoring the outcomes of decisions in social situations (105), which is in line with previous findings of NoGo P3 amplitude predicting more self-control during social decision-making (89). It is possible, therefore, that the reduced NoGo P3 amplitude and delayed NoGo P3 latency in the MDO group stem from a dysfunctional dorsal anterior or midcingulate cortex, which would be at least partly in line with the “frontal dysfunction hypothesis” of antisocial behavior (52, 53), depending on how “frontal” is defined. It must be mentioned, however, that research has also localized the source of the NoGo P3 to the left orbitofrontal cortex, which is inconsistent with a primary neural generator for the NoGo P3 in the MCC/dACC (106). There are also indications of a regional dissociation between the N2 and P3 components, with the former associated with anterior MCC activity and the latter with posterior MCC activity (33). Thus, the lack of NoGo N2 effects in the current study may reflect a more posterior MCC dysfunction in MDOs in the current study, although this remains speculative until further examined.

As expected, the MDOs were heterogeneous both in terms of mental disorders, criminal history, and pharmacological treatment (Figure 1C, Table 1). Like the majority of male MDOs in Sweden (54), most MDOs in the current study were diagnosed within the schizophrenia spectrum. Notwithstanding the wealth of research demonstrating P3 amplitude reductions in individuals characterized by trait disinhibition, reduced P3 amplitudes and prolonged P3 latencies have also been reported and replicated in individuals with schizophrenia [for reviews, see (107, 108)]. Again, research investigating the NoGo P3 component specifically is less common, but a recent study comparing NoGo P3 amplitude and latency in patients with schizophrenia and healthy controls found evidence of reduced NoGo P3 amplitude, but not delayed NoGo P3 latency, in patients compared to controls (86). Thus, it is difficult to disentangle whether the observed differences in NoGo P3 amplitude and latency in the current study were related to trait disinhibition, schizophrenia spectrum disorders, or other mental disorders. For instance, previous research (109), using a visual oddball task and a 30-item Trait Disinhibition scale from the full ESI (ESI_DIS_), found a negative correlation between ESI_DIS_ and P3b amplitude (*r* ~ −0.18) in a large (*N* = 419) sample of adult twins. Similarly, recent research found that higher ESI-BF_DIS_ scores predicted lower NoGo P3 amplitude (β = −0.16), after controlling for gender effects, in a sample (*N* = 142) of undergraduate students (20). In contrast, the correlations between ESI-BF_DIS_ score and NoGo P3 amplitude measures in the current study were practically zero. Our results may differ from previous findings due to several reasons, although perhaps most notably due differences in sample size and characteristics (MDOs plus healthy controls vs. only healthy participants). Still, recent research suggests that NoGo P3 latency may in fact be unrelated to schizophrenia (86). In the current study, we observed a positive and robust correlation between ESI-BF_DIS_ score and NoGo P3 latency (ρ = 0.19), suggesting that trait disinhibition may be associated with less efficient neural processing during outcome evaluation. This finding aligns well with the observed group differences in ESI-BF_DIS_ score and NoGo P3 latency, and warrants further research into the role of the NoGo P3 component as a transdiagnostic marker of trait disinhibition in MDOs.

Taken together, thus, it is possible that a dysfunctional MCC/dACC leads to inefficient neural processing during outcome evaluation, subsequently increasing the risk of behaviors characterized by trait disinhibition. However, several caveats, in addition to those already mentioned, must be taken into consideration. For instance, physically active, fit individuals demonstrate increased P3 amplitudes and reduced P3 latencies compared to sedentary individuals (110, 111), and previous studies have highlighted the poor physical state of Swedish inpatient MDOs (112). Furthermore, we did not assess any potential effects of mental fatigue, which has been shown to lead to lower NoGo P3 amplitudes (113, 114). Finally, MDOs' pharmacological treatment may also have affected our results. Most MDOs (74%) received some form of antipsychotic medication. Studies have shown that NoGo N2 amplitude is modulated by dopamine D1 receptors in the nigrostriatal system, while NoGo P3 amplitude is modulated by dopamine D2 receptors in the mesocorticolimbic dopamine system, such that higher receptor efficiency is associated with larger amplitudes and better response inhibition performance (31, 115). Typical antipsychotics act as dopamine D2 antagonists, while atypical antipsychotics are weaker dopamine D2 receptor antagonists, but also target other receptors, including dopamine D1 (116–118). In addition, 26% of MDOs in the current study received some form of antidepressant. Treatment using citalopram, a selective serotonin reuptake inhibitor commonly used as an antidepressant, has been demonstrated to result in NoGo P3 amplitude increases relative to placebo treatment (119). Any potential variability introduced by pharmacological treatment may, to some extent, be ameliorated using larger sample sizes in future studies.

### Clinical Implications

The results of this study may have several clinical implications. We found a high degree of both diagnostic and transdiagnostic trait disinhibition in the study's sample of male, violent, inpatient MDOs. Since the relationship between mental disorder and recidivism seems largely indirect, efforts aimed at reducing recidivism rates in MDOs should explicitly target behaviors characterized by trait disinhibition (3, 4). From a biobehavioral point of view, our findings could also be of importance for future studies on recidivism in MDOs. The long-term stability of the NoGo P3 makes it particularly suitable for use in clinical settings (120), and recent studies show that neurobiological data can be used to enhance the prediction of recidivism (121–123). In clinical practice, such models may be used to, for instance, direct treatment efforts to where they are most needed. Relatedly, we suggested that MDOs may have deficits in efficiently allocating enough cognitive resources during monitoring or evaluation of their behavior, which possibly can lead to impaired self-control in social decision-making (89). Thus, treatment aimed at improving MDOs' self-control in social contexts may be important. Other promising developments along these lines include the Reasoning and Rehabilitation for Mentally Disordered Offenders Programme (R&R2MP), which has been successful in decreasing disruptive behavior and improving rational problem-solving and attitudes toward violence in MDOs (124, 125), and recent work suggesting that Cognitive Remediation Training (CRT) may improve cognitive function in MDOs with schizophrenia or schizoaffective disorder (126). Finally, pharmacological treatment may also be an avenue worth exploring in more detail. Some encouraging progress has been made in individuals with ADHD, particularly on the effects of methylphenidate, a stimulant often used in ADHD treatment. Methylphenidate is thought to enhance the mechanisms involved in inhibiting automated response tendencies, particularly in the MCC/dACC (127). This effect might be related to modulation of the mesocorticolimbic dopamine system, since methylphenidate acts by prolonging dopamine availability in the synaptic cleft, leading to stronger dopamine D2 receptor activation (128). Studies in children with ADHD have found improved response inhibition and increased NoGo P3 amplitudes following methylphenidate treatment (129–131), and a study on adults with ADHD found that 6 weeks of methylphenidate treatment led to robust increases in inhibitory-related activation in the frontal cortex and the ACC (132). In contrast, another study found that symptomatic improvements following 6–8 weeks of methylphenidate treatment in a sample of adolescents with ADHD were related to reduced activity in, among others, the ACC (133). The authors speculate that methylphenidate treatment may have reduced the need for prefrontal inhibitory efforts, thereby leading to reduced prefrontal activation. Still, although the effects on NoGo P3 seem promising, methylphenidate has yet to be tested in MDO samples, possibly due to the fact that methylphenidate may induce psychotic symptoms.

### Strengths and Limitations

Some notable strengths of this study include the well-characterized sample of violent, inpatient MDOs, the robust, Bayesian statistical approach, and the open practices used. Studies on inpatient, violent MDOs are rare, in particular due to security concerns and difficulties in recruiting. Thus, while the limited and heterogeneous sample hinders the robustness and generalizability of our results, the current study takes an important step toward evaluating results of previous research in the domain of forensic mental health. Besides facilitating robustness by allowing the use of non-Gaussian likelihood functions, Bayesian statistics makes it possible to report genuine probability statements about the parameters of interest that, importantly, remain equally valid regardless of sample size (134), which is important in studies with smaller sample sizes. Finally, all computational resources used in the current study are publicly available at the Open Science Framework (https://osf.io/yscdh/) for anyone to review and reuse, and we urge other researchers to do the same.

One limitation, apart from the several caveats mentioned previously, was that the current study was not pre-registered, and all results should therefore be cautiously interpreted until replicated in independent samples. Furthermore, the inclusion of male, violent MDOs limits generalizability to non-violent and female MDOs. The bivariate mapping approach used in the current study is not optimal, since individual variation due to anxiousness, distractibility, or mental fatigue can affect task performance and lead to variance that is not specific to what the task is designed to measure (135). Unfortunately, no data on previous violent behavior (e.g., criminal records) or symptoms of mental disorder was available for control group participants. Future studies should consider incorporating such measures in order to increase the robustness of findings. Finally, a methodological limitation is the moderate reliability of the NoGo N2 latency observed in the current study. Previous research has found that the N2 ERP requires more trials than does the P3 in order to achieve similar levels of internal consistency (136). Although the average number of trials in the current study (mean *N* = 28) should be more than adequate, the number of trials ranged between 6 and 47 in the whole sample. Thus, it is possible that the number of trials were not sufficient for some participants, rendering NoGo N2 latency less reliable than the other measures. This could be ameliorated in future work by using a higher number of Go/NoGo trials.

### Summary and Directions for Future Research

To the best of our knowledge, this is the first study to demonstrate delayed NoGo P3 latency and attenuated NoGo P3 amplitude in inpatient, violent MDOs compared to healthy controls. With the above-mentioned limitations in mind, our findings are indicative of aberrant post-synaptic neurotransmission related to outcome monitoring or outcome evaluation in MDOs, possibly as a result of MCC/dACC dysfunction. Although in line with the “frontal dysfunction hypothesis” of antisocial behavior, further research using a larger MDO sample, preferably with EEG in conjunction with other neuroimaging techniques, is necessary to further elucidate potential MCC/dACC dysfunction in MDOs. Future research may also want to consider using other response inhibition tasks, such as the Stop-Signal Task, or social decision-making tasks, which might better reflect real-life scenarios of response inhibition. Composite measures and a structural modeling approach, more in line with the biobehavioral framework suggested by Venables et al. (12) and Patrick et al. (135), should also be considered. Such an approach requires larger sample sizes, which may be difficult to obtain in MDO populations, and multicenter studies are therefore recommended. Finally, although legislation concerning MDOs differs between countries, the current study represents an important step toward addressing the scarcity of neuroscientific research in inpatient MDOs, and sets the stage for future research that may want to investigate differences in NoGo P3 ERP between violent MDOs and non-violent psychiatric patients.

## Data Availability Statement

The raw data supporting the conclusions of this article will be made available by the authors, without undue reservation.

## Ethics Statement

The studies involving human participants were reviewed and approved by the regional ethics review board in Linköping, Sweden (2017/56-31, 239 2018/7-32, and 2018/321-32). The patients/participants provided their written informed consent to participate in this study.

## Author Contributions

CD, MW, and PA developed the study concept. ER contributed to the study design. Data collection and data analysis was performed by CD. CD performed EEG data preprocessing under supervision from ER. CD drafted the paper. MW, PA, ER, and MB provided critical revisions. All authors approved the final version of the paper for submission.

## Conflict of Interest

The authors declare that the research was conducted in the absence of any commercial or financial relationships that could be construed as a potential conflict of interest.
