# Supplementary material for: Trait Disinhibition and NoGo Event-Related Potentials in Violent Mentally Disordered Offenders and Healthy Controls
Source: Front Psychiatry. 2020 Dec 11;11:577491. doi: 10.3389/fpsyt.2020.577491 (PMC7759527; doi:10.3389/fpsyt.2020.577491)
Supplement: Supplementary file 1 [file Data_Sheet_1.PDF]

Supplementary Material

1 Effect Size Calculation

The population parameter estimated by a sample standardized mean difference, assuming common population standard deviation, is expressed as:

$$\delta = \frac{\mu_1 - \mu_2}{\sigma_*}$$

$$\sigma_* = \sigma_1 = \sigma_2 = \sigma$$

We can estimate δ using Hedges's *g* (often incorrectly labelled as Cohen's *d*):

$$g = \frac{M_1 - M_2}{S_p}$$

where s_p is an unbiased, pooled estimate of variance (which assumes homogeneity of population variance):

$$S_p = \sqrt{\frac{(n_1 - 1)S_1^2 + (n_2 - 1)S_2^2}{n_1 + n_2 - 2}}$$

However, *g* is a positively biased estimate of δ, although the bias is fairly small unless sample sizes are small (e.g., N < 20). Nonetheless, it is trivial to approximate the unbiased estimate of δ:

$$\hat{\delta} = g(1 - \frac{3}{4df_w - 1})$$

For additional details, please refer to (1), in particular pages 101-103.

2 Supplementary Tables and Figures

Supplementary Table 1. Overview of EEG data preprocessing results.

	Whole sample		Controls		Patients		Posterior estimates		
Measure	Mean ± SD	Range	Mean ± SD	Range	Mean ± SD	Range	Diff. [90% HDI]	$\hat{\delta}$ [90% HDI]	P_D
Number of bad channels interpolated	4.09 ± 4.07	0 - 16	5 ± 4.47	0 - 16	3.41 ± 3.69	0 - 11	-1.44 [-3.58, 0.59]	-0.36 [-0.89, 0.15]	88%
Number of ICAs zeroed out	7.94 ± 3.99	0 - 17	7.7 ± 3.99	2 - 17	8.11 ± 4.05	0 - 17	0.58 [-1.4, 2.63]	0.15 [-0.36, 0.66]	69%
Number of correct NoGo trials left after Autoreject	28.17 ± 9.88	6 - 47	28.25 ± 9.9	6 - 44	28.11 ± 10.04	9 - 47	-0.31 [-5.3, 4.73]	-0.03 [-0.52, 0.48]	54%

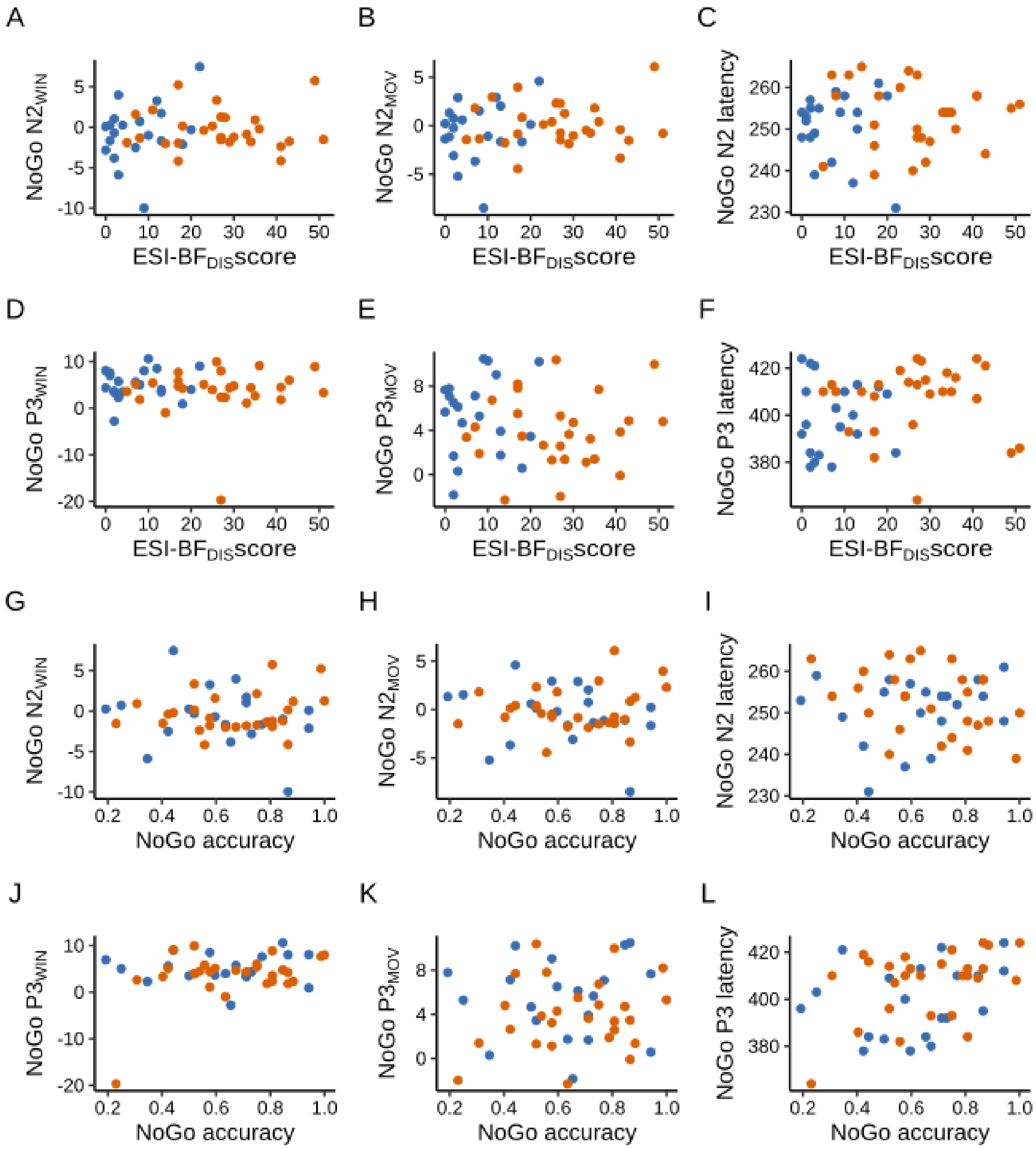

Supplementary Figure 1. Scatterplots showing bivariate relationships between ERP measures and (A-F) ESI-BF$_{DIS}$ scores and (G-L) NoGo accuracy. Blue dots represent controls, orange dots represent mentally disordered offenders.

References

1. Kline RB. *Beyond Significance Testing: Reforming Data Analysis Methods in Behavioral Research*. American Psychological Association (2004).
